# Training in Residency and Provision of Reproductive Health Services Among Family Medicine Physicians

**DOI:** 10.1001/jamanetworkopen.2023.30489

**Published:** 2023-08-23

**Authors:** Julia Strasser, Ellen Schenk, Qian Luo, Mandar Bodas, Olivia Anderson, Candice Chen

**Affiliations:** 1 Fitzhugh Mullan Institute for Health Workforce Equity, Department of Health Policy and Management, Milken Institute School of Public Health, George Washington University, Washington, DC

## Abstract

**Importance:**

Contraception and abortion services are essential health care, and family medicine (FM) physicians are an important part of the workforce providing this care. Residency could inform the reproductive health services FM physicians provide.

**Objective:**

To determine which residency training factors are associated with FM physicians’ provision of reproductive health services to Medicaid beneficiaries.

**Design, Setting, and Participants:**

This cross-sectional, population-based observational study of inpatient and outpatient FM physicians who completed residency between 2008 and 2018 and treated at least 1 Medicaid beneficiary in 2019 was conducted from November 2022 to March 2023. The study used 2019 American Medical Association Masterfile and Historical Residency file, as well as the 2019 Transformed Medicaid Statistical Information System claims.

**Exposures:**

Residency training in community-based or reproductive health-focused programs.

**Main Outcomes and Measures:**

The outcomes were providing the following to at least 1 Medicaid beneficiary in 2019: prescription contraception (pill, patch, and/or ring), intrauterine device (IUD) and/or contraceptive implant, and dilation and curettage (D&C). Odds of providing each outcome were measured using correlated random-effects regression models adjusted for physician, residency program, and county characteristics.

**Results:**

In the sample of 21 904 FM physician graduates from 410 FM residency programs, 12 307 were female (56.3%). More than half prescribed contraception to Medicaid beneficiaries (13 373 physicians [61.1%]), with lower proportions providing IUD or implant (4059 physicians [18.5%]) and D&C (152 physicians [.7%]). FM physicians who graduated from a Reproductive Health Education in Family Medicine program, which fully integrates family planning into residency training, had significantly greater odds of providing prescription contraception (odds ratio [OR], 1.23; 95% CI, 1.07-1.42), IUD or implant (OR, 1.79; 95% CI, 1.28-2.48), and D&C (OR, 3.61; 95% CI, 2.02-6.44). Physicians who completed residency at a Teaching Health Center, which emphasizes community-based care, had higher odds of providing an IUD or implant (OR, 1.51; 95% CI, 1.19-1.91).

**Conclusions and Relevance:**

In this cross-sectional study of FM physicians providing Medicaid service, characteristics of residency training including community-based care and integration of family planning training are associated with greater odds of providing reproductive health services. With growing reproductive health policy restrictions, providing adequate training in reproductive health is critical to maintaining access to care, especially for underserved populations.

## Key Points

**Question:**

What residency training factors are associated with family medicine (FM) physicians’ provision of reproductive health services to Medicaid beneficiaries?

**Findings:**

This cross-sectional observational study of 21 904 FM physicians found that several residency program characteristics were significantly associated with provision of common methods of contraception and dilation and curettage. Larger programs, programs with fully integrated family planning, and programs with an emphasis on community-based care produced graduates with higher odds of providing reproductive health services.

**Meaning:**

These findings suggest improving family planning and abortion training in family medicine programs could expand the workforce that can provide reproductive health services, thus improving access to care.

## Teaser

This cross-sectional study evaluates what residency training factors are associated with provision of reproductive health services among family medicine physicians.

## Introduction

Contraception and abortion services are essential health care, but many individuals face barriers in accessing this care. The workforce providing abortion and contraception care, which includes women’s health specialists, primary care physicians, and others, can impede or improve access. Although abortion care is often treated as a siloed issue, there is substantial overlap between the workforce providing abortion care, contraception care, and the full scope of reproductive health care. The same procedures, medications, and clinical skillset required to provide induced abortion are also used to manage early pregnancy loss, as well as other reproductive health indications. Although some primary care physicians provide the full scope of reproductive health services, many do not. Understanding more about the factors that are associated with providing these services, such as training, can help address gaps in access to care.

Family medicine (FM) physicians are an important part of the workforce providing contraception and abortion care, especially in populations that have limited access overall. In rural areas, women are more likely to see an FM physician than an obstetrician-gynecologist (OBGYN) for office-based well woman care.^1^ This may be due, in part, to the distribution of OBGYNs; 60% of US counties had an OBGYN or nurse-midwife in 2021, while more than 90% of counties had an FM physician.^2^ Our previous research found more than half of FM physicians provide prescription contraception, but fewer than 20% of those providing contraception care also provided implants or intrauterine devices (IUDs).^3^ In addition, while FM physicians can provide abortion or management of pregnancy loss, only a small number actually do so.^4^ For the Medicaid population, only 10% of primary care physicians who saw Medicaid patients provided implants or IUDs.^5^ Medicaid is particularly important for reproductive health care, covering nearly 20 million women of reproductive age and more than 40% of all US births.^6,7,8^ Although Medicaid coverage varies across states, Medicaid beneficiaries have also been shown to face a number of challenges in accessing care, including a lack of clinicians who accept Medicaid and longer wait times for appointments.^9,10,11,12^ Recent research has found contraceptive use among Medicaid beneficiaries varies significantly across states and counties.^13^

For physicians, residency training has been shown to imprint on future practice behaviors, including better maternal health outcomes, intensity of health care, and cost of care. The Teaching Health Center (THC) model of residency training focuses on community-based primary care. The THC program requires residency-sponsoring institutions to be either community-based ambulatory patient care centers or consortia that have a community-based ambulatory patient care center as a primary partner. Residents at THCs must meet all the same didactic and practical requirements as residents in hospital-sponsored programs, but these programs have prioritized outpatient primary care, often in rural and underserved settings.^14^ Although THC residencies are not the only residencies that are community-based (some FM residencies take place in community hospitals), the unique approach to graduate medical education calls for specific attention. THCs have been shown to produce FM physicians who are more likely to treat Medicaid patients, work in rural and underserved communities, and have a wider scope of practice than the general FM physician population.^15,16^

Some FM residency programs fall short in providing adequate training for reproductive health services. In a recent study of FM graduates, more than 20% reported that their residency training did not adequately prepare them for implant or IUD insertion, and only 40% reported providing these services.^17^ Just 16% reported adequate training for uterine aspiration/dilation and curettage (D&C) with less than 5% reporting providing these services.^17^

To address these gaps in training, some residency programs opt into the Reproductive Health Education in Family Medicine (RHEDI) program. The RHEDI program started in 2004 and integrates contraception and abortion training into participating FM residency programs. To meet RHEDI’s certification requirements, FM residency programs must include opt-out training for abortion, early pregnancy loss management, contraception, and other components of reproductive health care. There are currently 31 RHEDI residency programs in 17 states. Studies of RHEDI graduates have found they are more likely to provide IUD, implant, and abortion care compared with non-RHEDI trained FM physicians.^18,19,20^

One challenge to fully understanding the contraception and abortion workforce, including the role of residency training, has been the availability of reliable national level data.^21,22^ Although surveys and self-report can illuminate important aspects of the workforce, they can also be subject to reporting bias. This study uses national-level Medicaid claims data to examine the associations between FM physicians’ residency training and actual service provision of widely used methods of contraception and D&C.

## Methods

This study was approved by the George Washington University institutional review board. Because this is a study of secondary data, the study was exempt from the need for informed consent. The study followed the Strengthening the Reporting of Observational Studies in Epidemiology (STROBE) reporting guideline for observational studies.

### Data Sources

We used the American Medical Association (AMA) 2019 Masterfile and Historical Residency file to determine physician specialty, residency program details, and physician demographics. To identify which programs were THCs, we used a publicly available list from the Health Resources and Services Administration.^23^ To determine which programs were RHEDI programs, we used a proprietary list of programs provided to us by RHEDI program staff (personal communication, Erica Chong and Aleza Summit, November 2022).

We used 3 components of the 2019 Transformed Medicaid Statistical Information System (T-MSIS) data set: (1) Other Services file, (2) Pharmacy file, and (3) Annual Provider file to identify physicians’ service provision to Medicaid patients.^24^ Together, these files contain encounter data for physicians’ services (including *Current Procedural Terminology* [*CPT*], Healthcare Common Procedure Coding System codes, and *International Statistical Classification of Diseases and Related Health Problems, Tenth Revision [ICD-10]* codes), prescription claims data, National Provider Identifiers (NPI) for billing and servicing providers, and state of service.

We used 2015 to 2019 US Census Bureau American Community Survey (ACS) 5-year estimates for county-level demographics,^25^ and policy analyses by the Kaiser Family Foundation and the Guttmacher Institute to determine state-level Medicaid policies.^26,27^ State policies are characterized as having or not having 2 types of policies as of January 1, 2019: (1) Medicaid expansion status and (2) presence of Section 1115 waiver or State Plan Amendment for family planning services.

To determine the presence of an active NPI, we used the National Plan and Provider Enumeration System (NPPES). The NPPES is a national registry of health care clinicians which includes mailing and practice address, credentials, and specialty type; registration is required for any individual who conducts HIPAA-covered electronic transactions.^28^

### Study Population

To construct our sample, we used NPIs to merge data from the AMA Masterfile, NPPES, and T-MSIS. We included individuals who (1) had completed a US-based FM residency as their most recent residency program during the years 2008 to 2018, (2) were in active clinical practice, (3) treated at least 1 Medicaid beneficiary in calendar year 2019, and (4) practiced in a state with adequate claims data quality (43 states and Washington, DC were included; Arkansas, Delaware, Florida, Maine, Minnesota, New Hampshire, and Texas were excluded). We used a 10-year period to increase our sample size for more robust analysis, and we focused specifically on the years 2008 to 2018 to align with completing residency training at least 1 year before the 2019 claims data but not so long as to dampen the effect of residency training (eg, more than 10 years in the past). The eFigure in Supplement 1 shows the sample construction process.

### Outcome Measures

We constructed 5 measures of reproductive health services, identifying FM physicians who provided the following services to at least 1 Medicaid beneficiary in 2019: (1) prescription contraception (pill, patch, and/or ring) (2) IUD insertion and/or implant placement, and (3) dilation and curettage (D&C). For the contraceptive measures (1 and 2), we used a modified set of *CPT* codes and National Drug Codes (NDC) released by the Office of Population Affairs.^29^ For D&C, we included *CPT* codes for dilation and curettage (D&C) as well as dilation and evacuation (D&E), but as most procedures were D&C, we use the term D&C throughout to include both sets of procedures. See eTable 1 in Supplement 1 for a complete list of *CPT* codes.

### Statistical Analysis

To provide insight into the characteristics of graduates providing each outcome measure listed previously, descriptive analyses were performed for clinician, residency, practice, and state characteristics. Control variables include sex, years since residency graduation, degree type (MD, DO), practice location (rural vs nonrural), county-level characteristics, county-level physician-to-population ratio of OBGYNs, and state Medicaid policies; additional details are available in eTable 2 in Supplement 1.

We then estimated a correlated random-effects logistic model. One advantage of a correlated random-effects model is that it allows for estimation of both within-state and between-state variations in service provision. To construct the model, we followed a process outlined elsewhere in the literature to create variables according to whether they are constant at the state level or not.^30,31^ We then estimated a logistic regression with our control variables and a state random effect. Standard errors were clustered at the state level to account for heteroskedasticity. A 2-sided significance threshold of *P* = .025 was used. Stata MP, version 17 (StataCorp LLC) was used to conduct all analyses.^32^ We present the coefficients of the within-state and state policy effects here; full regression results including between-state effects can be found in eTable 4 in Supplement 1.

As a sensitivity analysis, we ran 2 additional regression series: (1) limiting our sample to physicians who saw at least 1 reproductive-age female Medicaid beneficiary and (2) limiting our sample to physicians who saw at least 10 reproductive-age female Medicaid beneficiaries. Results from sensitivity analyses (eTable 5 and eTable 6 in Supplement 1, respectively) show that our findings are robust to alternate model specifications and sample selection criterion. We therefore present results from our original model.

## Results

In our sample of 21 904 FM physicians, 12 307 were female (56.3%), 3491 practiced in rural counties (16.0%), and 11 492 had graduated from residency more than 5 years ago (52.5%); the full sample includes graduates of 410 FM residency programs. Table 1 presents descriptive characteristics of the physicians in the total sample and by reproductive service type.

**Table 1.** Characteristics of the Sample

Characteristic	Individuals, No. (%)			
	Full sample (N=21 904)	Pill, patch, or ring (n=13 373)	IUD or implant (n=4059)	D&C (n=152)
Sex				
Male	9597 (43.8)	5019 (37.5)	1164 (28.7)	39 (25.7)
Female	12 307 (56.2)	8354 (62.5)	2895 (71.3)	113 (74.3)
Practice in rural county	3491 (16.0)	2263 (16.9)	775 (19.1)	49 (32.2)
Years since residency program graduation				
>5	11 492 (52.5)	6694 (50.1)	1699 (41.1)	51 (33.6)
Degree and training type				
US trained, doctor of medicine	10 856 (49.6)	7146 (53.4)	2795 (68.9)	118 (77.6)
US trained, doctor of osteopathy	3007 (13.7)	2005 (15.0)	591 (14.6)	17 (11.2)
International medical graduate	8041 (36.7)	4222 (31.6)	673 (16.6)	17 (11.2)
RHEDI program graduate	1588 (7.3)	1095 (8.2)	582 (14.3)	49 (32.2)
Teaching health center graduate	740 (3.4)	439 (3.3)	266 (6.6)	11 (7.2)
Average yearly size of residency program				
1-5 Residents	3403 (15.5)	2007 (15)	487 (12)	10 (6.6)
6-10 Residents	11 363 (51.9)	6953 (52)	1987 (49)	58 (38.2)
≥11 Residents	7138 (32.6)	4413 (33)	1585 (39)	84 (55.3)
County level, mean (SD)^a^				
Non-Hispanic Asian American and Pacific Islander population	6.2 (7.3)	6.2 (7.3)	5.6 (6.6)	5.9 (7.5)
Non-Hispanic Black population	12.0 (13.4)	11.4 (12.9)	9.4 (12.1)	11.4 (14.4)
Hispanic population	15.9 (14.8)	16.8 (15.5)	14.9 (13.6)	18.0 (16.4)
Non-Hispanic American Indian or Alaskan Native or multiple race population	3.5 (5.0)	3.4 (5.1)	3.6 (4.4)	4.9 (9.5)
Non-Hispanic white population	62.4 (21.3)	62.3 (22.0)	66.5 (19.7)	59.9 (23.9)
Below poverty line	13.6 (4.9)	13.6 (4.9)	13.2 (4.6)	15.2 (5.8)
Female population aged 15-44 y	235 964 (430 259.2)	250 532 (457 121.7)	165 245 (284 711.0)	188 399 (349 572.6)
OBGYNs with at least 1 Medicaid beneficiary per 10 000 female population aged 15-44 y	6.2 (3.2)	6.09 (3.3)	6.3 (3.3)	5.8 (3.1)
State level				
Practice in Medicaid expansion state	16 261 (74.2)	10 181 (76.1)	2855 (70.3)	105 (69.0)
Practice in state with family planning waiver or SPA	13 524 (61.7)	7894 (59.0)	2481 (61.1)	103 (67.7)

Abbreviations: D&C, dilation and curettage; IUD, intrauterine device; OBGYN, obstetrics and gynecology; RHEDI, Reproductive Health Education in Family Medicine; SPA, state plan amendment.

^a^ County in which a clinician was located.

More FM physicians treated beneficiaries with prescription contraception compared with any other contraception method (13 373 physicians [61.1%]), followed by IUD insertion and/or implant placement (4059 physicians [18.5%]). Less than 1% provided D&C (152 physicians [0.7%]). There was substantial variation in the provision of services among the 410 programs in the sample. A low percentage of most residency programs’ graduates provided these services but the range was wide; some programs had 0% of graduates prescribing contraception and some had 100%. For IUD and/or implant, the percentage of graduates ranged from 0% to just more than 80%, and for D&C, most programs clustered at 0% (eTable 3 in Supplement 1).

Selected results from the correlated random-effects model are shown in Table 2. Female and younger physicians had significantly higher odds of providing all types of contraception and D&C services to the Medicaid population. Female physicians had 2.15 times the odds of prescribing the contraceptive pill, patch, or ring (95% CI, 2.01-2.29), 2.37 times the odds of providing IUD or implant (95% CI, 2.15-2.63), and 2.04 times the odds of providing D&C (95% CI, 1.33-3.13). Physicians who had graduated more than 5 years ago had 0.77 times the odds of prescribing the contraceptive pill, patch, or ring (95% CI, 0.72-0.83), 0.56 times the odds of providing IUD or implant (95% CI, 0.52-0.60), and 0.53 times the odds of providing D&C (95% CI, 0.39-0.73). All results were statistically significant.

**Table 2.** Selected Results for Odds of Providing Prescription Contraception, IUD and/or Implant, and D&C, 2019^a^

Characteristic	Pill, patch, and ring		IUD or implant		D&C	
	Odds ratio (95% CI)	*P* value	Odds ratio (95% CI)	*P* value	Odds ratio (95% CI)	*P* value
Sex						
Male	1 [Reference]	NA	1 [Reference]	NA	1 [Reference]	NA
Female	2.15 (2.01-2.29)	<.001	2.37 (2.15-2.63)	<.001	2.04 (1.33-3.13)	<.001
Years since residency program graduation						
≤5	1 [Reference]	NA	1 [Reference]	NA	1 [Reference]	NA
>5	0.77 (0.72-0.83)	<.001	0.56 (0.52-0.60)	<.001	0.53 (0.39-0.73)	<.001
RHEDI program graduate	1.23 (1.07-1.42)	.005	1.79 (1.28-2.48)	<.001	3.61 (2.02-6.44)	<.001
Residency at Teaching Health Center	1.05 (0.86-1.29)	.62	1.51 (1.19-1.91)	<.001	1.51 (0.59-3.83)	.39
Average yearly size of residency program						
1-5 Residents	1.02 (0.91-1.13)	.77	0.74 (0.60-0.90)	<.002	0.35 (0.17-0.73)	.005
6-10 Residents	1.06 (0.96-1.16)	.26	0.94 (0.86-1.03)	.16	0.65 (0.43-0.99)	.04
≥11 Residents	1 [Reference]	NA	1 [Reference]	NA	1 [Reference]	NA

Abbreviations: D&C, dilation and curettage; IUD, intrauterine device; NA, not applicable; RHEDI, Reproductive Health Education in Family Medicine.

^a^ Full regression models including control variables (medical degree type, rural vs nonrural practice location, county-level demographics, state-level policies) are available in eTable 4 in Supplement 1.

For our residency program characteristics of interest, we found higher odds of service provision among RHEDI graduates, THC graduates, and graduates of larger programs (Figure). RHEDI program graduates had significantly higher odds of providing prescription contraception (OR, 1.23; 95% CI, 1.07-1.42) and IUD or implant (OR, 1.79; 95% CI, 1.28-2.48). RHEDI graduates had 3.61 times the odds of providing D&C (95% CI, 2.02-6.44).

**Figure.**
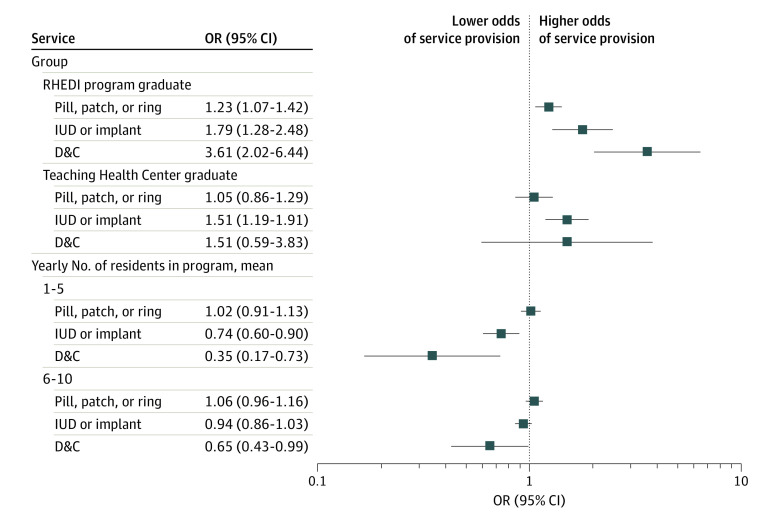
Association of Residency Program Type and Average Residency Size with Provision of Pill, Patch, and/or Ring, Intrauterine Device (IUD) and/or Implant, and Dilation and Curettage (D&C). Reference categories are graduates of non–Reproductive Health Education in Family Medicine programs, graduates of non–Teaching Health Center programs, and program sizes of 11 or fewer residents, respectively.

Physicians who completed their residency at a THC had higher odds of providing IUD or implant (OR, 1.51; 95% CI, 1.19-1.91). Results for prescription contraception and D&C were not statistically significant.

Compared with physicians who trained in larger programs (≥11 average residents per year), residents in smaller programs (1-5 residents) had significantly lower odds of providing IUD and/or implant (OR, 0.74; 95% CI, 0.60-0.90) and D&C (OR, 0.35; 95% CI, 0.17-0.73), while those in medium-sized programs (6-10 residents) had significantly lower odds of providing D&C (OR, 0.65; 95% CI, 0.43-0.99). Results were not statistically significant for prescription contraception. The full regression model and sensitivity analyses are available in eTable 4, eTable 5, and eTable 6 in Supplement 1.

## Discussion

Our cross-sectional analysis found that certain characteristics of residency training are associated with higher odds of contraception and D&C service provision among FM physicians who treat Medicaid beneficiaries. Specifically, RHEDI program and THC graduates were more likely to provide IUDs and/or implants, and graduates of RHEDI programs are more likely to provide both prescription contraception and D&C services as well. Despite the positive outcomes associated with graduates of these programs, RHEDI and THC residencies make up a small proportion of all FM residency programs across the US, and without expanded financial and structural support for these programs, the additional benefit of training in these environments will be not available to the entire field.

Consistent with the literature,^4^ our analysis found low levels of FM physician service provision across the different types of contraception and D&C, with only 19% of FM physicians providing IUDs and/or implants and less than 1% providing D&Cs for this population. FM physicians are the backbone of the workforce that cares for the Medicaid populations, and low rates of IUD, implant, and D&C provision may indicate lack of access to these services for Medicaid beneficiaries. Although low rates may reflect patient demand and/or preferences for certain methods,^33^ this study required a physician to provide only a single contraceptive service in an entire year to be considered a provider of that method. To improve access to care, FM physicians should provide the full scope of reproductive health services to all patients seeking them, a scenario that relies on access to adequate training in these services and exposure to underserved populations.

Female and younger physicians were more likely to provide all of our services of interest. This is consistent with previous research on the contraception workforce. Although these physician-level characteristics are immutable factors (a physician cannot change how old they are), they are important determinants of access to care because they indicate how the future of the workforce may take shape. In 2011, 34% of the FM physician workforce was female, and as of 2021, it had grown to 42% female.^34^ The future FM physician workforce may be more inclined to provide reproductive health services and to demand better training.

Training in these services is likely to become more difficult to obtain as policies go into effect that ban or limit them. In June 2022, the *Dobbs v Jackson Women’s Health Organization* Supreme Court decision removed federal protections for abortion, resulting in a wave of state-level restrictions and complete bans on abortion care. A concurring opinion on the *Dobbs* decision also stated that certain legal precedents, including those that grant the right to contraception,^35^ should be reconsidered, and there is now a distinct possibility that federal protections for contraception may be removed in the coming years. As the number of states banning or restricting abortion increases, the role of state policies will increase in residency training. Early reports suggest that a lower proportion of medical students in the class of 2023 selected residency training in states that ban abortion.^36^ Future research should continue to examine the role of the state of residency training in providing these services.

Although the data from this study are pre-*Dobbs*, they capture important baseline data on the interaction between residency training, reproductive health services, and care for underserved populations. As these essential health care services are increasingly politicized, the clinical environment is becoming increasingly hostile,^37,38^ but FM has yet to implement specific changes in light of the *Dobbs* decision. Strengthening training and accreditation requirements to fully integrate contraception and abortion clinical skills into FM residency would ensure a robust future workforce for this care. Without expanded training, limited access to learning contraception and pregnancy termination skills will likely result in a loss of this skillset over time. This, in turn, will result in limited access to care and worse outcomes, especially for populations that face higher rates of maternal morbidity and mortality.^39,40^

### Limitations

Although this article presents strong evidence for the role of training in FM physicians’ provision of contraception and abortion, some limitations must be noted. First, our findings only apply to the population covered by Medicaid and may not be generalizable to the full population. Second, we used large national data sets that have known limitations. With the T-MSIS data, we excluded 7 states from the analysis due to documented data quality issues.^41^ However, other published literature using T-MSIS data has followed similar data quality checks,^42^ and this study nonetheless provided analysis from 43 states and Washington, DC. We also note that there are documented limitations to the AMA Masterfile. NPIs of some recent graduates may not be up-to-date; we found that 319 physicians who graduated during the study period did not have an NPI associated with AMA Masterfile Records (1.4% of the total sample). Also, AMA Masterfile only reports Accreditation Council for Graduate Medical Education–accredited residency programs, resulting in a possible undercount of Doctors of Osteopathy. Third, we note that there may be a self-selection bias for RHEDI and THC programs, which may attract residents more likely to provide these services to underserved populations. Additionally, abortion services are not covered by federal Medicaid dollars, except under limited circumstances, and this study likely captures information primarily on physicians providing D&C for management of pregnancy loss, ectopic pregnancy, and indications other than induced abortion.

## Conclusion

Residency training is associated with the FM physicians’ provision of reproductive health services. In light of rapidly shifting policies that include, but are not limited to, the *Dobbs* decision, the outcomes of training future physicians are critically associated with maintaining access to care, especially for underserved populations.

## Notes

Supplement 1. **eTable 1. **Outcome Definitions and CPT/ICD-10 Codes **eFigure. **Study Sample Flow Diagram **eTable 2. **Covariate Description and Data Source **eTable 3. **Additional Descriptive Statistics **eTable 4. **Full Regression Results **eTable 5. **Regression Results for Seeing at Least 1 Woman of Reproductive Age (15-44 years) **eTable 6. **Regression Results for Seeing at Least 10 Women of Reproductive Age (15-44 years)

Supplement 2.
Data Sharing Statement
