# Supplementary material for: Training in Residency and Provision of Reproductive Health Services Among Family Medicine Physicians
Source: JAMA Netw Open. 2023 Aug 23;6(8):e2330489. doi: 10.1001/jamanetworkopen.2023.30489 (PMC10448301; doi:10.1001/jamanetworkopen.2023.30489)
Supplement: Supplement 1. — eTable 1. Outcome Definitions and CPT/ICD-10 Codes eFigure. Study Sample Flow Diagram eTable 2. Covariate Description and Data Source eTable 3. Additional Descriptive Statistics eTable 4. Full Regression Results eTable 5. Regression Results for Seeing at Least 1 Woman of Reproductive Age (15-44 years) eTable 6. Regression Results for Seeing at Least 10 Women of Reproductive Age (15-44 years) [file jamanetwopen-e2330489-s001.pdf]

Supplemental Online Content

Strasser J, Schenk E, Luo Q, Bodas M, Anderson O, Chen C. Training in residency and provision of reproductive health services among family medicine physicians. *JAMA Netw Open*. 2023;6(8):e2330489. doi:10.1001/jamanetworkopen.2023.30489

eTable 1. Outcome Definitions and CPT/ICD-10 Codes
eFigure. Study Sample Flow Diagram
eTable 2. Covariate Description and Data Source
eTable 3. Additional Descriptive Statistics
eTable 4. Full Regression Results
eTable 5. Regression Results for Seeing at Least 1 Woman of Reproductive Age (15-44 years)
eTable 6. Regression Results for Seeing at Least 10 Women of Reproductive Age (15-44 years)

© 2023 Strasser J et al. *JAMA Network Open.*

eTable 1. Outcome Definitions and *CPT*/*ICD*-10 Codes

Outcome	Definition	Codes
Providing prescription contraception	At least one beneficiary with a visit for the contraceptive pill, patch, and/or ring	See Office of Population Affairs Contraception Provision Measures[1]
Providing IUD insertion and/or contraceptive implant	At least one beneficiary with a visit for IUD insertion and/or at least one beneficiary with a visit for contraceptive implant placement	IUD<br>CPT: 58300, S4981, S4989, J7302, J7300, J7296, J7297, J7301, J7298<br>Implant<br>CPT: 11981, 11983, J7307, J7306<br>ICD-10: For 11981 and 11983 only: Z30.017, Z30.018, Z30.019, Z30.40, Z30.46, Z30.49, Z30.8, Z30.9
Providing termination of pregnancy	At least one beneficiary with a visit for procedural abortion, including dilation and curettage, dilation and evacuation, and other surgical procedures	CPT: 59820, 59812, 59821, 58120, 59840, 59841

[1] US Dept of Health and Human Services; Office of the Assistant Secretary for Health; Office of Population Affairs. "Contraceptive Provision Measures." Accessed October 4, 2022. https://opa.hhs.gov/claims-data-sas-program-instructions.

© 2023 Strasser J et al. *JAMA Network Open.*

eFigure. Study Sample Flow Diagram

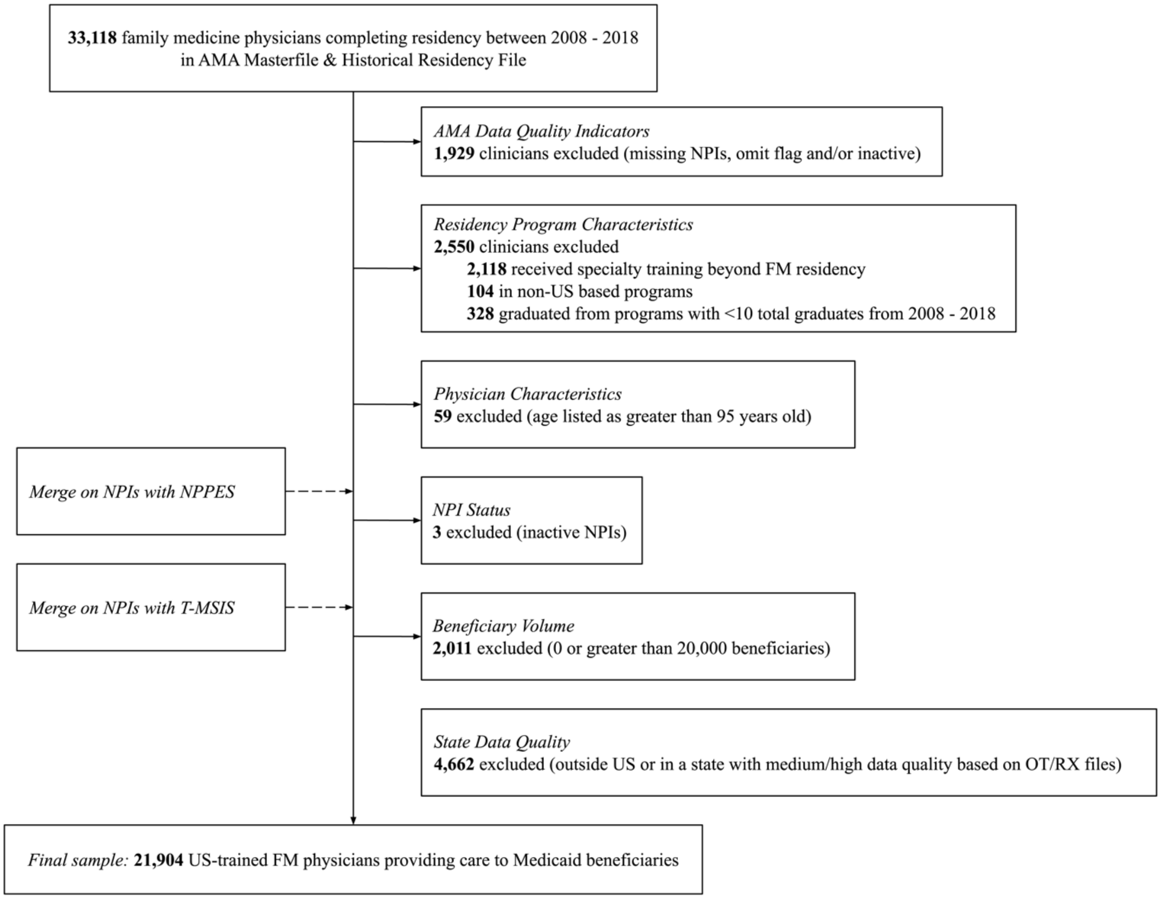

© 2023 Strasser J et al. *JAMA Network Open.*

eTable 2. Covariate Description and Data Source

Variable	Type	Description/notes	Data source
**Provider characteristics**			
Sex	Binary	Male (reference); female	AMA Masterfile
Practice in rural county	Binary	Urban (reference; RUCC 1, 2, 3); rural (RUCC 4, 5, 6, 7, 8, 9)	2013 USDA RUCC
Years since residency program graduation	Binary	5 or fewer years since residency program graduation (reference); greater than 5 years since residency program graduation; difference between graduation year and 2019	AMA Historical Residency File
Degree type	Categorical	US trained, MD (reference); US trained, DO; international medical graduate	AMA Masterfile
**Residency program characteristics**			
RHEDI program graduate	Binary	Graduated from Non-RHEDI program (reference); graduated from RHEDI program (graduated at least one year after program became RHEDI program)	AMA Historical Residency File
Teaching Health Center graduate	Binary	Non-teaching health center residency (reference); completed residency at teaching health center	AMA Historical Residency File
Average yearly size of residency program	Ordinal	1-5 residents; 6-10 residents; 11+ residents (reference)	AMA Historical Residency File
**County characteristics**			
Percent race/ethnicity	Continuous	Non-Hispanic white (reference); Non-Hispanic Black; Non-Hispanic Asian American and Pacific Islander; Hispanic, Non-Hispanic American Indian and Alaska Native, Other, or multiple race population;	2015 – 2019 American Community Survey 5-year estimates
Percent below poverty line	Continuous	Population below poverty line	2015 – 2019 ACS 5-years estimates
Female population, reproductive age	Continuous	County female population aged 15-44	2015 – 2019 ACS 5-year estimates
OBGYNs per capita providing Medicaid services per 10,000 female population of reproductive age	Continuous	OBGYNs (specialty from NPPES) who treated at least one Medicaid beneficiary (any service) per female population aged 15-44	T-MSIS claims data; 2019 NPPES; 2015 – 2019 ACS 5-year estimates
**State Medicaid policies**			
Medicaid expansion	Binary	Medicaid expansion as of January 1, 2019	Kaiser Family Foundation
Medicaid family planning eligibility expansions	Binary	Family planning benefits through waiver or state plan amendment (SPA) as of January 1, 2019. Family planning waivers are time-limited expansions for eligibility for Medicaid coverage of family planning services, while SPAs are a permanent change.	Guttmacher Institute

© 2023 Strasser J et al. *JAMA Network Open.*

eTable 3. Additional Descriptive Statistics

**Characteristic, No. (%)**	Full Sample (N = 21904)	Pill/Patch/Ring (N = 13373)	IUD/Implant (N = 4059)	D&C (N = 152)
Individual level				
Physicians seeing Medicaid beneficiaries				
1-10 beneficiaries	1326 (6.1)	138 (1.0)	0 (0)	0 (0)
11-50 beneficiaries	2214 (10.1)	839 (6.3)	21 (.5)	3 (2.0)
51+ beneficiaries	18364 (83.8)	12396 (92.7)	4038 (99.5)	149 (98.0)
Physicians seeing reproductive age (15-44) female Medicaid beneficiaries [a]				
1-10 beneficiaries	2655 (12.1)	640 (4.8)	6 (.2)	0 (0)
11-50 beneficiaries	6131 (28.0)	3119 (23.3)	381 (9.4)	6 (4.0)
51+ beneficiaries	12458 (56.9)	9610 (71.9)	3672 (90.5)	146 (96)
Program level				
Residency program graduates (physicians) providing services as a proportion of total individual program graduates				
Median (IQR)	-	49.3 (33.3 - 62.5)	8.9 (3.6 - 18.8)	0 (0 - 0)
Minimum	-	0	0	0
Maximum	-	100	81.2	10.8
Proportion of programs (N = 410) with at least one residency program graduate (physicians) providing services	-	403 (98.3)	326 (79.5)	34 (8.3)

[a] Numbers may not add up to full sample N, and percentages may not add up to 100 because some physicians did not see any females of reproductive age or rounding.

© 2023 Strasser J et al. *JAMA Network Open.*

eTable 4. Full Regression Results

	Odds Ratio (95% Confidence Interval)		
N = 21,904	Pill/Patch/Ring	IUD/Implant	D&C
**Individual level**			
Sex			
Male	1 [Reference]	1 [Reference]	1 [Reference]
Female	2.15** (2.01 - 2.29)	2.37** (2.15 - 2.63)	2.04** (1.33 - 3.13)
Practice county rurality			
Urban	1 [Reference]	1 [Reference]	1 [Reference]
Rural	1.11 (0.95 - 1.30)	0.90 (0.73 - 1.10)	2.32** (1.37 - 3.93)
Years since residency program graduation			
5 or less	1 [Reference]	1 [Reference]	1 [Reference]
Greater than 5	0.77** (0.72 - 0.83)	0.56** (0.52 - 0.60)	0.53** (0.39 - 0.73)
Degree and training type			
US trained, doctor of medicine	1 [Reference]	1 [Reference]	1 [Reference]
US trained, doctor of osteopathy	1.00 (0.90 - 1.13)	0.70** (0.61 - 0.79)	0.75 (0.46 - 1.23)
International medical graduate	0.55** (0.50 - 0.61)	0.33** (0.28 - 0.40)	0.27** (0.16 - 0.47)
RHEDI program graduate	1.23** (1.07 - 1.42)	1.79** (1.28 - 2.48)	3.61** (2.02 - 6.44)
Residency at Teaching Health Center	1.05 (0.86 - 1.29)	1.51** (1.19 - 1.91)	1.51 (0.59 - 3.83)
Average yearly size of residency program			
1-5 residents	1.02 (0.91 - 1.13)	0.74** (0.60 - 0.90)	0.35** (0.17 - 0.73)
6-10 residents	1.06 (0.96 - 1.16)	0.94 (0.86 - 1.03)	0.65* (0.43 - 0.99)
11+ residents	1 [Reference]	1 [Reference]	1 [Reference]
**County level** [a]			
Percent Non-Hispanic AAPI population	1.00 (0.99 - 1.01)	0.99* (0.97 - 1.00)	1.01 (0.99 - 1.03)
Percent Non-Hispanic Black population	1.00 (1.00 - 1.01)	1.01 (0.99 - 1.02)	1.02 (0.99 - 1.04)
Percent Hispanic population	1.01** (1.00 - 1.01)	1.00 (0.99 - 1.01)	1.02 (0.99 - 1.04)
Percent Non-Hispanic AIAN, Other, or multiracial population	0.99* (0.98 - 1.00)	0.97** (0.95 - 0.98)	1.00 (0.97 - 1.03)
Percent Non-Hispanic white population	1 [Reference]	1 [Reference]	1 [Reference]
Percent below poverty line	1.01 (0.99 - 1.02)	1.02 (0.99 - 1.04)	1.03 (0.98 - 1.08)

© 2023 Strasser J et al. *JAMA Network Open.*

Female population aged 15-44 (log)	0.94 (0.88 - 1.01)	0.92 (0.84 - 1.01)	0.96 (0.76 - 1.20)
OBGYNs treating at least one Medicaid beneficiary per 10,000 female population aged 15-44	0.97** (0.96 - 0.98)	1.01 (0.99 - 1.03)	0.95 (0.89 - 1.00)
**State level**			
Medicaid expansion	1.30 (0.87 - 1.94)	1.30 (0.46 - 3.70)	1.23 (0.47 - 3.24)
Family planning waiver or SPA	0.74 (0.40 - 1.37)	1.26 (0.83 - 1.92)	1.51 (0.75 - 3.04)
State Means			
**Individual level**			
Female	0.92 (0.05 - 17.89)	0.18 (0.00 - 169.15)	0.10 (0.00 - 37.11)
Practice in rural county	1.35 (0.24 - 7.48)	0.33 (0.02 - 5.78)	3.66 (0.06 - 212.76)
Greater than 5 years since residency program graduation	0.03 (0.00 - 1.62)	0.00* (0.00 - 0.70)	0.00 (0.00 - 15.92)
Degree and training type			
US trained, doctor of osteopathy	15.00 (0.76 - 296.48)	2.30 (0.01 - 973.57)	5.00 (0.01 - 3,994.49)
International medical graduate	0.86 (0.09 - 7.88)	0.01** (0.00 - 0.07)	0.17 (0.00 - 6.71)
RHEDI program graduate	2.02 (0.05 - 77.29)	8.82 (0.46 - 168.29)	0.32 (0.00 - 25.23)
Residency at Teaching Health Center	0.97 (0.03 - 32.08)	6.19 (0.06 - 614.51)	14.78 (0.17 - 1,253.96)
1-5 residents	0.29 (0.03 - 2.47)	0.15 (0.01 - 3.40)	0.04 (0.00 - 2.24)
**County level** [a]			
Percent Non-Hispanic AAPI population	0.99 (0.96 - 1.01)	1.00 (0.96 - 1.04)	1.01 (0.94 - 1.08)
Percent Non-Hispanic Black population	0.99 (0.97 - 1.02)	0.98 (0.94 - 1.01)	1.01 (0.97 - 1.06)
Percent Hispanic population	1.01 (0.96 - 1.06)	0.99 (0.95 - 1.02)	1.00 (0.95 - 1.04)
Percent Non-Hispanic AIAN, Other, or multiple race population	1.00 (0.98 - 1.01)	0.98 (0.95 - 1.01)	1.05* (1.00 - 1.10)
Percent below poverty line	1.02 (0.97 - 1.08)	0.96 (0.88 - 1.05)	0.99 (0.85 - 1.15)
Female population aged 15-44 (log)	1.04 (0.58 - 1.85)	1.27 (0.73 - 2.18)	1.63 (0.77 - 3.44)
OBGYNs treating at least one Medicaid beneficiary per 10,000 female population aged 15-44	1.11 (0.90 - 1.37)	1.29 (0.86 - 1.93)	1.09 (0.81 - 1.46)

Abbreviations: IUD, intrauterine device; D&C, dilation and curettage; RHEDI, Reproductive Health Education in Family Medicine; AAPI, Asian American and Pacific Islander; AIAN, American Indian or Alaskan Native; OBGYN, obstetrics and gynecology; SPA, state plan amendment
** p<0.01, * p<0.05
[a] County in which a clinician was located

© 2023 Strasser J et al. *JAMA Network Open.*

eTable 5. Regression Results for Seeing at Least 1 Woman of Reproductive Age (15-44 years)

Odds Ratio (95% Confidence Interval)			
N = 21,366	Pill/Patch/Ring	IUD/Implant	D&C
**Individual level**			
Sex			
Male	1 [Reference]	1 [Reference]	1 [Reference]
Female	2.21** (2.06 - 2.36)	2.38** (2.15 - 2.63)	2.04** (1.33 - 3.12)
Practice county rurality			
Urban	1 [Reference]	1 [Reference]	1 [Reference]
Rural	1.12 (0.96 - 1.32)	0.90 (0.73 - 1.11)	2.33** (1.38 - 3.95)
Years since residency program graduation			
5 or less	1 [Reference]	1 [Reference]	1 [Reference]
Greater than 5	0.80** (0.74 - 0.85)	0.57** (0.53 - 0.61)	0.54** (0.40 - 0.74)
Degree and training type			
US trained, doctor of medicine	1 [Reference]	1 [Reference]	1 [Reference]
US trained, doctor of osteopathy	1.01 (0.90 - 1.13)	0.70** (0.62 - 0.80)	0.76 (0.46 - 1.24)
International medical graduate	0.54** (0.50 - 0.59)	0.33** (0.28 - 0.40)	0.27** (0.16 - 0.47)
RHEDI program graduate	1.22** (1.06 - 1.42)	1.78** (1.28 - 2.47)	3.62** (2.03 - 6.47)
Teaching Health Center program graduate	1.07 (0.88 - 1.31)	1.52** (1.20 - 1.93)	1.51 (0.59 - 3.83)
Average yearly size of residency program			
1-5 residents	1.02 (0.91 - 1.14)	0.74** (0.61 - 0.90)	0.35** (0.17 - 0.74)
6-10 residents	1.04 (0.94 - 1.15)	0.93 (0.85 - 1.02)	0.65* (0.43 - 0.99)
11+ residents	1 [Reference]	1 [Reference]	1 [Reference]
**County level**			
Percent Non-Hispanic AAPI population	1.00 (0.99 - 1.01)	0.99* (0.97 - 1.00)	1.01 (0.99 - 1.03)
Percent Non-Hispanic Black population	1.00 (1.00 - 1.01)	1.01 (1.00 - 1.02)	1.02 (0.99 - 1.04)
Percent Hispanic Population	1.01** (1.00 - 1.01)	1.00 (0.99 - 1.01)	1.01 (0.99 - 1.04)
Percent Non-Hispanic AIAN, Other, or multiracial population	0.99* (0.98 - 1.00)	0.97** (0.95 - 0.98)	1.00 (0.97 - 1.03)
Percent Non-Hispanic white population	1 [Reference]	1 [Reference]	1 [Reference]
Percent below poverty line	1.01 (0.99 - 1.02)	1.02 (0.99 - 1.04)	1.03 (0.98 - 1.08)
Female population aged 15-44 (log)	0.95 (0.88 - 1.01)	0.92 (0.84 - 1.02)	0.96 (0.77 - 1.21)
OBGYNs treating at least one Medicaid beneficiary per 10,000 female population aged 15-44	0.97** (0.96 - 0.98)	1.01 (0.99 - 1.03)	0.95 (0.89 - 1.00)

© 2023 Strasser J et al. *JAMA Network Open.*

**State level**			
Medicaid expansion	1.29 (0.86 - 1.95)	1.29 (0.44 - 3.77)	1.27 (0.48 - 3.34)
Family planning waiver or SPA	0.72 (0.39 - 1.34)	1.23 (0.81 - 1.87)	1.49 (0.75 - 2.95)
State Means			
**Individual level**			
Female	1.12 (0.06 - 20.73)	0.16 (0.00 - 117.44)	0.06 (0.00 - 19.14)
Practice in rural county	1.49 (0.25 - 8.83)	0.29 (0.01 - 5.99)	3.74 (0.07 - 209.95)
Greater than 5 years since residency program graduation	0.03 (0.00 - 2.37)	0.00 (0.00 - 1.40)	0.00 (0.00 - 9.57)
Degree and training type			
US trained, doctor of osteopathy	14.50 (0.71 - 297.69)	1.49 (0.00 - 746.59)	3.94 (0.01 - 2,701.31)
International medical graduate	0.84 (0.09 - 8.00)	0.01** (0.00 - 0.07)	0.19 (0.01 - 6.57)
RHEDI program graduate	2.38 (0.06 - 88.56)	10.67 (0.63 - 181.32)	0.35 (0.01 - 23.81)
Residency at Teaching Health Center	0.90 (0.03 - 31.52)	6.89 (0.07 - 656.46)	17.29 (0.20 - 1,516.64)
Average yearly size of residency program			
1-5 residents	0.31 (0.04 - 2.66)	0.14 (0.01 - 3.55)	0.04 (0.00 - 2.31)
6-10 residents	0.90 (0.34 - 2.38)	0.15 (0.01 - 2.09)	0.00** (0.00 - 0.07)
**County level** [a]			
Percent Non-Hispanic AAPI population	0.98 (0.96 - 1.01)	1.00 (0.96 - 1.04)	1.01 (0.94 - 1.08)
Percent Non-Hispanic Black population	1.00 (0.97 - 1.02)	0.98 (0.94 - 1.01)	1.02 (0.97 - 1.06)
Percent Hispanic Population	1.01 (0.96 - 1.06)	0.99 (0.95 - 1.02)	1.00 (0.95 - 1.04)
Percent Non-Hispanic AIAN, Other, or multiracial population	0.99 (0.98 - 1.01)	0.98 (0.95 - 1.01)	1.05* (1.00 - 1.10)
Percent below poverty line	1.02 (0.97 - 1.08)	0.96 (0.88 - 1.04)	0.98 (0.84 - 1.14)
Female population aged 15-44 (log)	1.05 (0.58 - 1.91)	1.24 (0.71 - 2.15)	1.63 (0.77 - 3.46)
OBGYNs treating at least one Medicaid beneficiary per 10,000 female population aged 15-44	1.11 (0.89 - 1.38)	1.28 (0.86 - 1.89)	1.09 (0.82 - 1.45)

Abbreviations: IUD, intrauterine device; D&C, dilation and curettage; RHEDI, Reproductive Health Education in Family Medicine; AAPI, Asian American and Pacific Islander; AIAN, American Indian or Alaskan Native; OBGYN, obstetrics and gynecology; SPA, state plan amendment

** p<0.01, * p<0.05

[a] County in which a clinician was located

© 2023 Strasser J et al. *JAMA Network Open.*

eTable 6. Regression Results for Seeing at Least 10 Women of Reproductive Age (15-44 years)

	Odds Ratio (95% Confidence Interval)		
N = 18,915	Pill/Patch/Ring	IUD/Implant	D&C
**Individual level**			
Sex			
Male	1 [Reference]	1 [Reference]	1 [Reference]
Female	2.45** (2.27 - 2.64)	2.44** (2.20 - 2.71)	2.06** (1.35 - 3.13)
Practice county rurality			
Urban	1 [Reference]	1 [Reference]	1 [Reference]
Rural	1.17 (0.98 - 1.39)	0.91 (0.74 - 1.14)	2.38** (1.41 - 4.02)
Years since residency program graduation			
5 or less	1 [Reference]	1 [Reference]	1 [Reference]
Greater than 5	0.83** (0.77 - 0.89)	0.59** (0.55 - 0.63)	0.57** (0.41 - 0.78)
Degree and training type			
US trained, doctor of medicine	1 [Reference]	1 [Reference]	1 [Reference]
US trained, doctor of osteopathy	1.02 (0.89 - 1.17)	0.70** (0.62 - 0.81)	0.79 (0.48 - 1.29)
International medical graduate	0.47** (0.43 - 0.52)	0.33** (0.27 - 0.39)	0.28** (0.16 - 0.48)
RHEDI program graduate	1.22* (1.03 - 1.46)	1.77** (1.25 - 2.51)	3.59** (2.02 - 6.37)
Residency at Teaching Health Center	1.07 (0.84 - 1.37)	1.56** (1.22 - 1.99)	1.51 (0.60 - 3.76)
Average yearly size of residency program			
1-5 residents	1.04 (0.93 - 1.17)	0.74** (0.61 - 0.91)	0.36** (0.17 - 0.76)
6-10 residents	1.05 (0.97 - 1.15)	0.93 (0.85 - 1.02)	0.65* (0.43 - 0.97)
11+ residents	1 [Reference]	1 [Reference]	1 [Reference]
**County level** [a]			
Percent Non-Hispanic AAPI population	1.00 (0.99 - 1.02)	0.99* (0.97 - 1.00)	1.01 (0.99 - 1.03)
Percent Non-Hispanic Black population	1.00 (1.00 - 1.01)	1.01 (1.00 - 1.02)	1.02 (0.99 - 1.04)
Percent Hispanic population	1.01** (1.00 - 1.01)	1.00 (0.99 - 1.01)	1.02 (0.99 - 1.04)
Percent Non-Hispanic AIAN, Other, or multiracial population	0.99* (0.98 - 1.00)	0.97** (0.95 - 0.98)	1.00 (0.97 - 1.03)
Percent Non-Hispanic white population			
Percent below poverty line	1.00 (0.98 - 1.02)	1.01 (0.99 - 1.04)	1.03 (0.98 - 1.08)
Female population aged 15-44 (log)	0.98 (0.90 - 1.06)	0.94 (0.85 - 1.04)	0.98 (0.78 - 1.23)

© 2023 Strasser J et al. *JAMA Network Open.*

OBGYNs treating at least one Medicaid beneficiary per 10,000 female population aged 15-44	0.96** (0.95 - 0.98)	1.01 (0.99 - 1.03)	0.95 (0.89 - 1.00)
**State level**			
Medicaid expansion	1.29 (0.80 - 2.06)	1.28 (0.45 - 3.61)	1.21 (0.45 - 3.23)
Family planning waiver or SPA	0.67 (0.34 - 1.33)	1.22 (0.81 - 1.84)	1.48 (0.73 - 3.00)
State Means			
**Individual level**			
Female	5.24 (0.16 - 166.35)	0.25 (0.00 - 253.91)	0.10 (0.00 - 43.26)
Practice in rural county	3.15 (0.33 - 29.80)	0.37 (0.02 - 6.37)	3.50 (0.06 - 192.91)
Greater than 5 years since residency program graduation	0.07 (0.00 - 8.44)	0.00* (0.00 - 0.55)	0.00 (0.00 - 16.04)
Degree and training type			
US trained, doctor of osteopathy	23.62 (0.58 - 958.69)	2.10 (0.01 - 849.58)	4.57 (0.01 - 3,518.76)
International medical graduate	0.49 (0.04 - 5.77)	0.01** (0.00 - 0.05)	0.17 (0.00 - 7.07)
RHEDI program graduate	1.99 (0.03 - 139.99)	8.62 (0.46 - 162.08)	0.28 (0.00 - 24.34)
Residency at Teaching Health Center	0.47 (0.01 - 26.92)	5.99 (0.06 - 586.74)	14.65 (0.15 - 1,480.81)
Average yearly size of residency program			
1-5 residents	0.32 (0.03 - 3.41)	0.19 (0.01 - 4.32)	0.05 (0.00 - 2.80)
6-10 residents	0.86 (0.27 - 2.76)	0.15 (0.01 - 2.10)	0.00** (0.00 - 0.08)
**County level** [a]			
Percent Non-Hispanic AAPI population	0.98 (0.96 - 1.01)	1.01 (0.97 - 1.05)	1.01 (0.95 - 1.08)
Percent Non-Hispanic Black population	1.01 (0.97 - 1.04)	0.98 (0.95 - 1.02)	1.02 (0.97 - 1.07)
Percent Hispanic Population	1.02 (0.96 - 1.07)	0.99 (0.95 - 1.03)	1.00 (0.95 - 1.05)
Percent Non-Hispanic AIAN, Other, or multiracial population	1.00 (0.98 - 1.02)	0.98 (0.95 - 1.01)	1.05 (1.00 - 1.10)
Percent below poverty line	1.02 (0.96 - 1.08)	0.96 (0.88 - 1.05)	0.99 (0.85 - 1.15)
Female population aged 15-44 (log)	1.17 (0.61 - 2.26)	1.29 (0.75 - 2.23)	1.59 (0.76 - 3.35)
OBGYNs treating at least one Medicaid beneficiary per 10,000 female population aged 15-44	1.05 (0.83 - 1.33)	1.25 (0.83 - 1.89)	1.07 (0.80 - 1.43)

Abbreviations: IUD, intrauterine device; D&C, dilation and curettage; RHEDI, Reproductive Health Education in Family Medicine; AAPI, Asian American and Pacific Islander; AIAN, American Indian or Alaskan Native; OBGYN, obstetrics and gynecology; SPA, state plan amendment
** p<0.01, * p<0.05
[a] County in which a clinician was located

© 2023 Strasser J et al. *JAMA Network Open.*
