# Supplementary material for: Training in Residency and Provision of Reproductive Health Services Among Family Medicine Physicians
Source: JAMA Netw Open. 2023 Aug 23;6(8):e2330489. doi: 10.1001/jamanetworkopen.2023.30489 (PMC10448301; doi:10.1001/jamanetworkopen.2023.30489)
Supplement: Supplement 2. — Data Sharing Statement [file jamanetwopen-e2330489-s002.pdf]

Data Sharing Statement

Strasser. Training in Residency and Provision of Reproductive Health Services among Family Medicine Physicians. *JAMA Netw Open*. Published August 23, 2023. doi:10.1001/jamanetworkopen.2023.30489

Data
Data available: No

Additional Information
Explanation for why data not available: We use identified claims data, which is highly sensitive and cannot be shared.
